# Supplementary figures and images for: Changes in local mineral homeostasis facilitate the formation of benign and malignant testicular microcalcifications
Source: eLife. 2025 Apr 25;13:RP95545. doi: 10.7554/eLife.95545 (PMC12029210; doi:10.7554/eLife.95545)

Figure 1-F

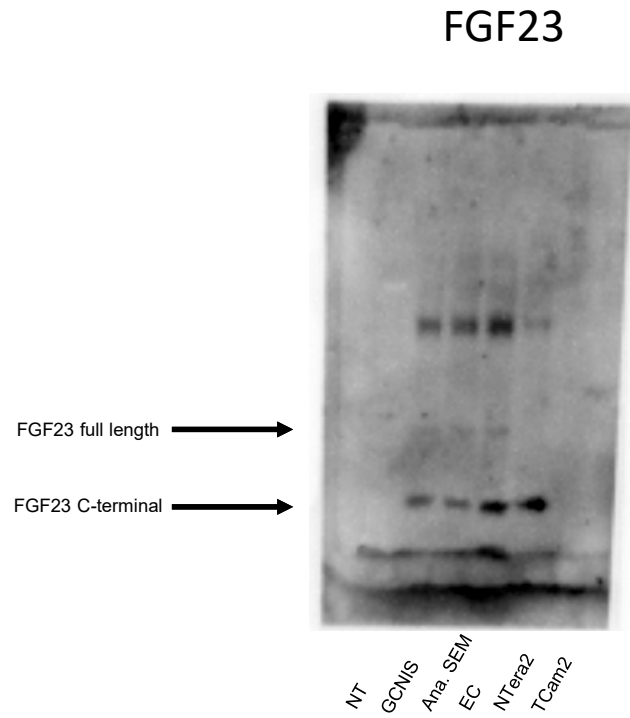

Figure 1-F

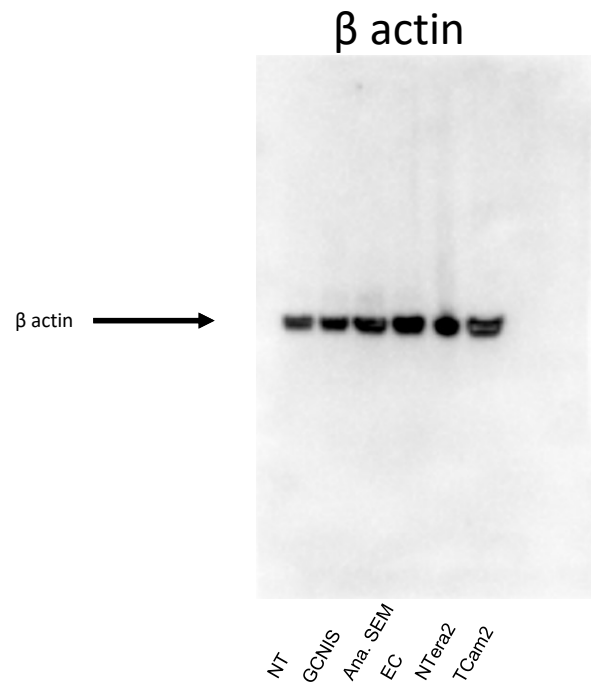

Supplement: Figure 2—source data 3. [file elife-95545-fig2-data3.zip › Original western blots for Figure 2,indicating the relevant bands/Figure 2-F.pdf]
