# Supplementary material for: Changes in local mineral homeostasis facilitate the formation of benign and malignant testicular microcalcifications
Source: eLife. 2025 Apr 25;13:RP95545. doi: 10.7554/eLife.95545 (PMC12029210; doi:10.7554/eLife.95545)
Supplement: Supplementary file 1. [file elife-95545-supp1.docx]

## Supplementary file 1

List of primary antibodies used for IHC staining

| Antibody | Catalog no. | Manufacturer | Dilution | Buffer | RRID |  |
| --- | --- | --- | --- | --- | --- | --- |
| Osteocalcin | sc-30044 | Santa Cruz biotechnology | 1:50 / 1:100 | CIT | AB_2064902 |  |
| DMP1 | sc-73633 | Santa Cruz biotechnology | 1:100 | TEG | AB_2292808 |  |
| FGF23 | sc-16849 | Santa Cruz biotechnology | 1:75 / 1:200 | TEG | AB_2104631 |  |
| FGFR1 | ab10646 | Abcam | 1:500 | TEG | AB_297367 |  |
| Klotho | sc-22220 | Santa Cruz biotechnology | 1:100 / 1:200 | TEG | AB_2131919 |  |
| MAGE | Gift from Prof. Spagnoli | Non-commercial | 1:500 | TEG | N.A. |  |
| NPT2a | sc-33928 | Santa Cruz biotechnology | 1:200 | TEG | AB_2254836 |  |
| OCT4 | sc-5279 | Santa Cruz biotechnology | 1:50 / 1:100 | TEG | AB_628051 |  |
| OPN | sc-73631 | Santa Cruz biotechnology | 1:100 | CIT | AB_2194995 |  |
| ALP | M7191 | Abcam | 1:100 | CIT | AB_2226182 |  |
| RUNX2 | sc-10758 | Santa Cruz biotechnology | 1:200 / 1:400 | TEG | AB_2184247 |  |
| SOX9 | AB5535 | Merck Millipore | 1:400 | TEG | AB_2239761 |  |

N.A. Not available

## 
