# Supplementary material for: Changes in local mineral homeostasis facilitate the formation of benign and malignant testicular microcalcifications
Source: eLife. 2025 Apr 25;13:RP95545. doi: 10.7554/eLife.95545 (PMC12029210; doi:10.7554/eLife.95545)
Supplement: Supplementary file 2. [file elife-95545-supp2.docx]

## Supplementary file 2

List of primers used for qRT-PCR

| Gene | Forward primer | Reverse primer |  |  |
| --- | --- | --- | --- | --- |
| Human | | |  |  |
| *ALPI* | TACACGTCCATCCTGTACGG | CTCGCTCTCATTCACGTCTGG |  |  |
| *ALPL* | ATGGGATGGGTGTCTCCACA | CCACGAAGGGGAACTTGTC |  |  |
| *ALPP* | CACCTGTGGGACTTGAGGAC | TTTGGCTCTCGACCAGTGTC |  |  |
| *ALPP2* | GGCATCATCCCAGTTGAGGAG | GCACATGCTTGTCTACACTGTAT |  |  |
| *B2M* | ATCCAATCCAAATGCGGCATC | AGTATGCCTGCCGTGTGAAC |  |  |
| *BGLAP* | CACTCCTCGCCCTATTGGC | GTGGTCAGCCAACTCGTCA |  |  |
| *FGF23* | CACCTGCAGATCCACAAGAA | TAATCACCACAAAGCCAGCA |  |  |
| *GAPDH* | TCAACGACCACTTTGTCAAGC | GGTGGTCCAGGGGTCTTACTC |  |  |
| *NANOG* | TGATTTGTGGGCCTGAAGAAAA | GAGGCATCTCAGCAGAAGACA |  |  |
| *POU5F1* | GACTCCTCGGTCCCTTTCC | CAAAAACCCTGGCACAAACT |  |  |
| *SLC34A1* | CCCTCAGGTCCTACACAGGAT | GGAGCAGACGAAGAGGTAGAG |  |  |
| *SLC34A2* | CTGAGGCACCTGTAACCAAGA | GGAGCACACGAAAAAGTAGAGA |  |  |
|  |  |  |  |  |
| Mouse | | |  |  |
| Abcc6 | GCATCGTTCAGGCTCGAGTG | CGAGGAGCGCCTGGAGTTAC |  |  |
| *Alpl* | CACGGCCATCCTATATGGTAA | GGGCCTGGTAGTTGTTGTGA |  |  |
| *B2m* | CGAGACATGTGATCAAGCATCA | TATTGCTCAGCTATCTAGGATAT |  |  |
| *Bglap* | CTGACCTCACAGATCCCAAGC | TGGTCTGATAGCTCGTCACAAG |  |  |
| *Cyp24a1* | ACACTGGCAGAGTACCACAAG | TCCAGGGTTTGATCTCTAGCC |  |  |
| *Cyp27b1* | GCACAGTTTACGTTGCCGAC | CGTTAGCAATCCGCAAGCA |  |  |
| *Enpp1* | AAGCGCTTACACTTCGCTAAAAG | TGATGGATTCAACGCAAGTTG |  |  |
| *Runx2* | AGAGTCAGATTACAGATCCCAGG | TGGCTCTTCTTACTGAGAGAGG |  |  |
| *Slc20a1* | TCATCATTGCATTTGTCTTGGCA | CGGATGGTTTCGCTCACTTTG |  |  |
| *Slc34a1* | GACAATGCCATCCTATCCAACC | GAGCCCATGATGATCGGAATG |  |  |
| *Slc34a2* | GACTGAACTGCTACCCTCCTA | ACCATTTGATCCCAGTGTCCT |  |  |

ds to 100 µm.
